# Comparison of laparoscopy-assisted surgery and laparotomy for treating locally advanced distal gastric antral cancer

**DOI:** 10.3892/etm.2013.1199

**Published:** 2013-07-03

**Authors:** FA FANG, FENG HAN, YIN-LU DING, HAI-JIANG WANG

**Affiliations:** 1 Department of Gastrointestinal Surgery, The Affiliated Tumor Hospital of Xinjiang Medical University, Urumqi, Xinjiang 830000, P.R. China; 2 Department of Emergency, The Affiliated Tumor Hospital of Xinjiang Medical University, Urumqi, Xinjiang 830000, P.R. China; 3 Department of General Surgery, The Second Hospital of Shandong University, Jinan, Shandong 250033, P.R. China

**Keywords:** gastric antrum cancer, laparoscopy-assisted surgery, laparotomy, cumulative survival rate, controlled clinical trials

## Abstract

The aim of this study was to investigate the safety, feasibility and mid-term results of laparoscopy-assisted surgery in the treatment of locally advanced gastric antral cancer. The clinical data of 50 patients who received laparoscopy-assisted surgery (Group A) and 62 patients who were treated by conventional laparotomy (Group B) from August 2009 to January 2011 were retrospectively analyzed. The surgical incision length, the volume of blood loss, the intestinal function recovery time, the postoperative complications, the postoperative 1- and 3-year cumulative survival rates and the average survival time in the two groups were observed. The results of the two groups were compared using the χ^2^ test for the enumeration data, a t-test for the numerical data and a Wilcoxon rank sum test for the skewed data. In addition, the Kaplan-Meier method of single factor analysis was utilized to comwpare the 1- and 3-year cumulative survival rates, as well as the average survival time of the two groups. The results indicated that the duration of surgery for Group A was significantly longer compared with that of Group B (P<0.05); however, the incision length and the volume of intraoperative blood loss in Group A were significantly smaller compared with those of Group B (P<0.01). Furthermore, in Group A, the recovery of intestinal function was more rapid and the time spent in hospital was shorter. However, between Groups A and B, the respective number of dissected lymph nodes (16.3 and 17.2), 1-year survival rates (86.0 and 88.6%) and 3-year survival rates (52.6 and 53.7%) were not significantly different (P<0.05). The results indicate that laparoscopy-assisted surgery is a safe approach for the treatment of locally advanced gastric antral cancer and has beneficial treatment effects. Laparoscopy-assisted surgery is advantageous compared with laparotomy, due to the smaller incision length and reductions in intraoperative blood loss, invasiveness, postoperative recovery time and the number of complications.

## Introduction

Gastric cancer is one of the most common malignant tumors, with the fourth highest incidence rate and the second highest mortality rate among the malignant types of cancer. It also has the highest incidence rate among the digestive tract cancers (1,2,3). Surgery is considered to be the only radical treatment method for gastric cancer. Due to an abundant gastric blood supply, complex anatomical section and lymphatic metastasis pathway, the anastomosis is difficult to operate on. The D2 radical operation for gastric cancer during the progression period is even more challenging. The surgery under laparoscope requires the physician to have a great experience in open surgery and skilled in laparoscopy techniques. The indications of laparoscopy-assisted D2 radical operation for gastric cancer in progressed stage remain the topics of controversy. The most important question is whether the gastric cancer surgery under laparoscope is able to achieve the radical cure. The D2 radical resection of gastric cancer consists of at least the three aspects: i) complete resection of the primary foci and surrounding tissues and organs with a sufficiently wide margin; ii) complete dissection of the gastric lymph nodes; iii) complete elimination of shed cancer cells in the abdominal cavity. The laparoscopy-assisted radical surgery for gastric cancer must conform to these rules, therefore a long-term survival may be assured and the advantage of minimally invasive surgery may be maximized. The laparoscope has a favorable local amplifying effect and is able to clearly visualize the blood vessels, nerves and fascia. The laparoscope is able to guarantee a higher precision for local operation and treatment of large vessels. The laparoscopy-assisted surgery is superior. Goh *et al* were the first to implement the laparoscopy-assisted D2 radical gastrectomy in advanced cases of gastric cancer in 1997 (4). The minimal invasiveness of the surgery is the predominant advantage when compared with traditional laparotomy. However, the safety, feasibility and prognosis of laparoscopy-assisted surgery have been the focus of debate. In this study, we retrospectively analyzed the clinical data of 50 patients receiving laparoscopic treatment (Group A) and 62 patients receiving conventional laparotomy (Group B), at The Affiliated Tumor Hospital of Xinjiang Medical University (Urumqi, China) from August 2009 to January 2011. The surgical incision length, volume of blood loss, postoperative recovery rate and complications, and the cumulative survival rates were compared between the two groups of patients, to investigate the advantages of laparoscopy in the treatment of locally advanced gastric antral cancer.

## Patients and methods

### Patients

The present study involved 112 patients with locally advanced gastric antral cancer, who received gastrointestinal surgery at our hospital from August 2009 to December 2010. The 112 cases comprised 52 males and 60 females (age, 33–77 years), 98 of whom were of Han ethnicity and 14 of whom were of an ethnic minority. In accordance with the willingness of the patients to undergo surgery, the patients were divided into two groups according to the surgical approach. These were Group A (50 cases), which comprised patients receiving laparoscopy, and Group B (62 cases), which comprised patients undergoing laparotomy. A general comparison of the patients in the two groups revealed no significant differences (P>0.05 for all parameters; Table I). The study protocol was approved by the ethics committee of Xinjiang Medical University (Urumqi, China), and written informed consent was obtained from each participant prior to data collection.

### Inclusion and exclusion criteria

The inclusion criteria for laparoscopy-assisted surgery were as follows: pathologically confirmed adenocarcinoma; preoperative clinical tumor stage T2–T3; the absence of extensive peritoneal implantation metastasis and lung, liver and bone metastasis according to preoperative clinical investigations, including chest X-ray, thoracoabdominal cavity computed tomography (CT) scan and a tumor marker test; and the absence at a general physical checkup of other factors that indicated that the patient was unsuitable for surgery. The exclusion criteria comprised: multiple primary gastric tumors; distant metastasis discovered during surgery; cases requiring emergency surgery for acute pyloric obstruction; and cases unsuitable for laparoscopy for other reasons.

### Methods

**Figure 1:**
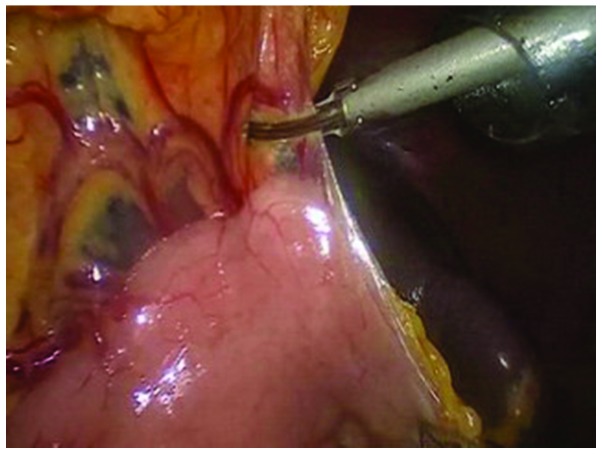
An ultrasound knife separated the anterior lobe of the transverse mesocolon, resulting in a free gastrocolic ligament.

**Figure 2:**
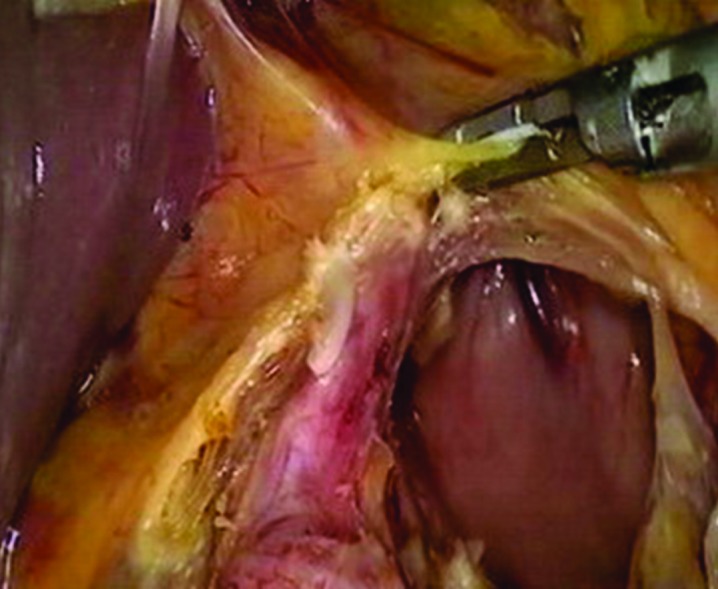
An ultrasound knife separated the anterior lobe of the transverse mesocolon resulting in a free right gastroepiploic vein.

**Figure 3:**
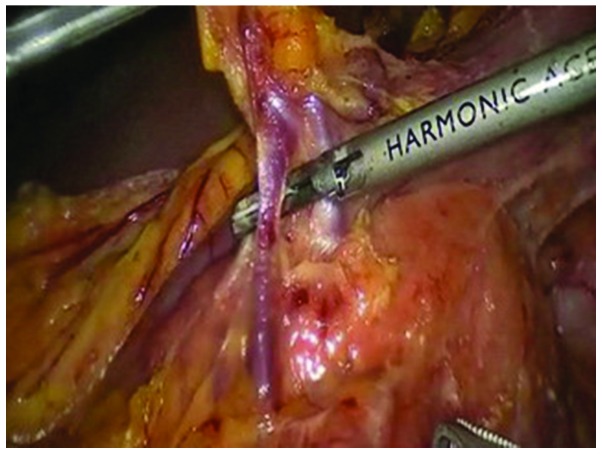
Cleaning of the group 12a lymph nodes.

**Figure 4:**
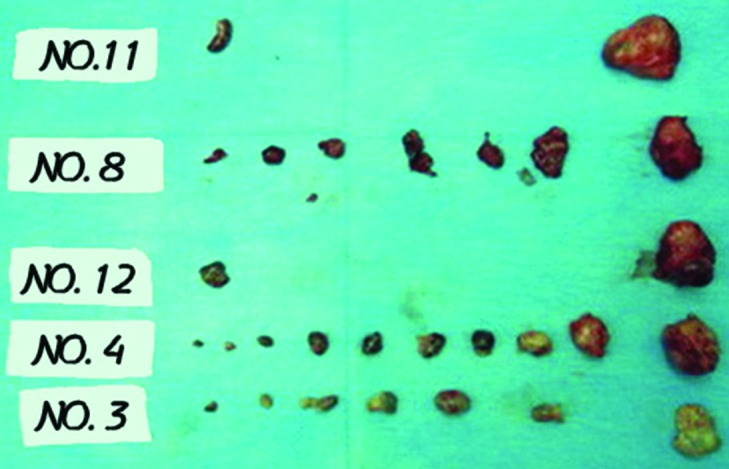
Intraoperative cleaning of the lymph nodes.

The same group of surgeons performed the surgical procedures in the two groups. The laparoscopy-assisted surgery required five incisions to be made in the patient. The pneumoperitoneum was established through an infraumbilical puncture, with a pressure of 12–14 mmHg. A 10-mm trocar was inserted through the infraumbilical route. The intraoperative principles for laparoscopy-assisted surgery included: i) application of D2 radical gastrectomy in all patients, ii) en bloc resection of the cancerous tumor and the surrounding tissues; and prevention of iatrogenic tumor spread. During the surgery, contact between the forceps and the tumor, as well as squeezing of the tumor, were prohibited. To prevent cancer implantation in the peritoneal wall incisions, there was no direct clamping of the tumor, and the tumor was removed through the protective ring. In addition, following removal, fluorouracil implants were distributed in the peritoneal cavity, after washing with saline. The protective ring was subsequently removed, and the incisions were washed for a second time. The pneumoperitoneum was relieved and the casing was removed. An ultrasound knife (HARMONIC ACE36E, Johnson & Johnson, New Brunswick, NJ, USA) was used to separate the anterior lobe of the transverse mesocolon, with laparoscopic assistance (Fig. 1). The perigastric blood vessels were next treated as follows: the left gastric artery, right gastric artery, right gastroomental vessels and right gastroomental vessels were clipped by biological vascular clips and other small blood vessels were closed by ultrasonic scalpel (Fig. 2). The lymph nodes were dissected as described previously (5), (Figs. 3 and 4). Of the patients receiving laparoscopy, 32 were treated with the Billorth I method and 18 were treated with the Billorth II method (6). For patients undergoing laparotomy, the periumbilical incision was made on the middle upper abdomen for traditional D2 radical gastrectomy.

### Follow-up

The follow-up included re-examination during hospitalization and as an outpatient, and regular telephone surveys for discharged patients who had received radical gastrectomy. The medical records of the patients were also evaluated for 14–30 months following the surgery (average duration, 18 months). A chest X-ray, an abdominal B ultrasound and the detection of carcinoembryonic antigen (CEA) were performed every month in the first six months following the surgery, and a fibergastroscopy was performed every 4–6 months. The follow-up period ended in April 2012. The patient follow-up rate was 93.75% (105/112). A case was lost according to the last follow-up calculation. Lost cases and fatalities that were not due to cancer were evaluated by statistical analysis for censored data processing requirements.

### Statistics

SPSS software, version 13.0 (SPSS, Inc., Chicago, IL, USA) was used for statistical analysis of the data. The results of Groups A and B were compared using the χ^2^ test for enumeration data, a t-test for numerical data and a Wilcoxon rank sum test for skewed data. In addition, the Kaplan-Meier method of single factor analysis was utilized to compare the 1- and 3-year cumulative survival rates, as well as the average survival time, between the two groups. P<0.05 was considered to indicate a statistically significant difference.

## Results

In Group A, 46 of the 50 patients underwent successful surgery with complete tumor resection and the median number of dissected lymph nodes per patient was 16.3. Four patients (8%) in Group A were transferred to the laparotomy procedure; three patients had severe adhesions due to obesity and one patient demonstrated hematoma and blood oozing due to low levels of blood coagulation. In Group B, the surgery was successful for all patients, the tumors were completely resected and the median number of dissected lymph nodes per patient was 17.2. No significant difference was identified in the number of dissected lymph nodes between Groups A and B (P>0.05).

The time taken for the gastrointestinal function to recover and the incidence of postoperative complications are shown in Table II. In Group A, the complications observed were: fat liquefaction at the incision, lung infection, anastomotic stoma stenosis, urinary tract infection, subcutaneous emphysema and lymphatic leakage (one case of each). In Group B, one case of each of small bowel obstruction, intra-abdominal hemorrhage, peritoneal infection and lymphatic leakage occurred; along with two cases of each of delayed gastric emptying, lung infection and urinary tract infection, and eight cases of fat liquefaction at the incision. Following the surgery, the gastrointestinal function recovery time of Group A was shorter than that of Group B (3.12±0.82 vs. 3.8±1.31 days; P<0.05), and the postoperative hospital stay of Group A was significantly shorter than that of Group B (18.94±7.81 vs. 23.61±9.02 days, respectively; P<0.05; Table II). The incidence of complications in Group A was significantly lower than that of Group B (6/50 vs. 18/62, respectively; P<0.05; Table II).

**Figure 5:**
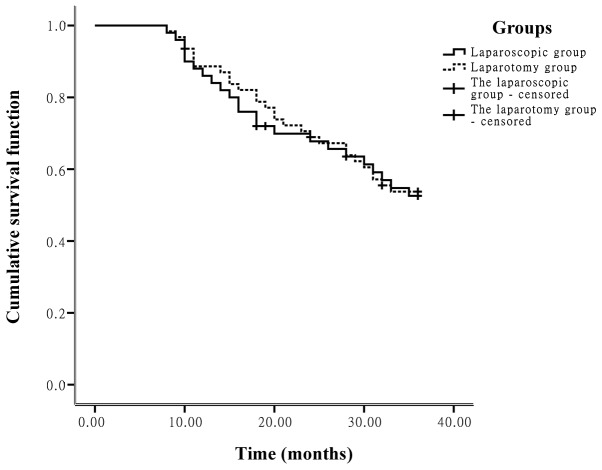
Cumulative survival rate.

Kaplan-Meier univariate analysis showed that in Groups A and B, the 1-year (86.0 and 88.6%, respectively) and 3-year cumulative survival rates (52.6 and 53.7%, respectively), and average survival times (28.13 and 28.65 months, respectively), were not statistically significantly different (P>0.05; Fig. 5).

## Discussion

The rapid development of minimally invasive surgery has facilitated a new approach to surgical treatment. Laparoscopic cholecystectomy has become a gold standard treatment, while laparoscopic radical resection is now recognized as an effective technique for colorectal cancer. This novel technique has also been applied to radical surgery for other types of tumors. However, the application of laparoscopic gastrectomy is limited due to the numerous gastric blood vessels, the levels of anatomical structure, the complex lymph node metastasis pathway and the presence of anastomosis. In 1994, Kitano *et al* reported the first use of the radical gastrectomy technique (7). Subsequently, Kitano *et al* described 116 cases of early-stage gastric cancer who received laparoscopy-assisted radical gastrectomy (8). The cases were followed up for an average of 45 months, and no cases of tumor recurrence or cancer implantation in the incisions were identified. The present study indicated that the number of dissected lymph nodes and the surgical margin of the groups receiving either laparoscopy-assisted surgery or laparotomy were similar (Table II), which was concordant with the results of previous studies (9–12). This is likely due to the fact that the tips of the laparoscope assisted the radical gastrectomy for gastric cancer, achieving a similar radical effect to that of conventional open surgery.

The duration of the surgery is an important indicator when evaluating a novel surgical technique. In the present study, the duration of the laparoscopy-assisted surgery was longer compared with that of the laparotomy. This may have been due to the fact that laparoscopy-assisted surgery is a more complex procedure than laparotomy, as it involves several abdominal regions and the surgical area is difficult to access. The cooperation between the surgeon and the assistants was suboptimal, which added to the surgical difficulty; and the laparoscopic technique takes time to master (13,14). Tokunaga *et al* determined that following professional training in laparoscopic gastrectomy, the duration of the surgery was decreased to marginally longer than that of the laparotomy, which indicates that physicians who are skilled at performing the laparotomy require additional experience prior to becoming skilled at conducting laparoscopic surgery (15). We propose that with an increasing number of patients undergoing laparoscopy-assisted surgery, the improvement in surgical skills may reduce the duration of the surgery to that of laparotomy.

The introduction of an ultrasound knife may decrease the volume of bleeding and the difficulty of tissue separation experienced in laparoscopic surgery. It may also produce less surgical smoke and carbon compared with an electric knife during surgery (16,17). However, how the ultrasound knife is used directly impacts the hemostatic effect. It will cause bleeding if the blood vessels are severed when the blood has not completely clotted; for areas with numerous blood vessels, if only part of the blood vessels are clamped, then the blood vessel severing will also cause bleeding. A frequently used method is to clean the field of vision with an aspirator, while clamping the bleeding points with a ultrasonic knife, set to a slow mode, to stop the bleeding (18–22). Varela *et al* revealed that the mean volume of intraoperative blood loss in patients receiving laparoscopy was significantly lower than that of patients undergoing laparotomy (138 vs. 57 ml, respectively) (23). The present study identified that the length of the incision, intraoperative blood loss, postoperative ventilation time, duration of hospitalization and the incidence of complications in the laparoscopic group were decreased compared with those in the laparotomy group. These advantages of laparoscopic gastrectomy are in accordance with the results of previous studies (24,25).

Implantation metastasis of the tumor in the incisions and peritoneal cavity is a significant disadvantage of laparoscopic surgery. However, whether the pressure difference in the CO_2_ pneumoperitoneum results in the shedding and implantation of tumor cells remains controversial. Certain scholars consider that the pressure of the CO_2_ pneumoperitoneum is not directly related to tumor metastasis (26,27). The present study involved a follow-up of 50 patients who underwent laparoscopic gastrectomy, in which one patient with implantation metastasis in the peritoneal cavity was identified shortly following the surgery. This may have been due to the following: i) the direct implantation of the shedded tumor cells; ii) trocar incision injury and leakage of CO_2_ along the trocar; iii) atomization of the tumor cells; and iv) the effect of artificial pneumoperitoneum on cellular immunity (28–31). Furthermore, when the number of tumor cells reaches a certain level, the cells escape from the collection pore due to the pressure difference in the CO_2_ pneumoperitoneum. A number of the cells adhere to the incisions or the incision margins, resulting in cancer implantation in the incisions, which is known as the ‘chimney effect’ (32). Therefore, tumor-free technology is particularly important in laparoscopy, to reduce implantation metastasis of the tumor in the incisions. In the present study, relevant measures were taken, such as strictly complying with the tumor-free principle (33,34), protecting the incisions when removing the tumor specimens, soaking and rinsing prior to the abdominal closure, and killing residual tumor cells by the intraperitoneal application of fluorouracil implants. These measures are important for reducing the risk of tumor implantation.

All patients receiving the laparoscopy-assisted surgery face the possibility of transferring to laparotomy, which limits the application of laparoscopic surgery. The reduction of the transferal rate is a key issue. Dulucq *et al* described eight patients who received laparoscopic total gastrectomy and 11 patients who underwent laparoscopic subtotal gastrectomy, among which there were no cases that were transferred to laparotomy (35). Pugliese *et al* investigated 48 cases who underwent laparoscopic total gastrectomy and subtotal gastrectomy, where only one patient was transferred to laparotomy due to a large tumor size (36). Shimizu *et al* revealed that eight out of 100 cases (8%) were transferred to laparotomy (37). In the present study, the transferal rate in the laparoscopy group was 8%, and postoperative complications occurred in three of the four patients who were transferred. The reason may be that the surgeon did not fully understand the indications of transferring to laparotomy, and failed to transfer in a timely manner, which extended the surgery time and affected the postoperative recovery. However, patients with combined cardiopulmonary diseases may benefit from the advantages of laparoscopic surgery, as it is minimally invasive. In practice, we propose that transferal to laparotomy should be considered when the following conditions are observed: i) a large, advanced-stage tumor, which has extensively invaded the surrounding tissues; ii) perigastric major blood vessels that are encapsulated by the tumor or metastatic lymph nodes; iii) a loss of normal anatomical spaces; iv) obesity and extensive adhesions; v) suspected metastases to substantial organs with poor classification and local invasion during surgery (which are observable, but not palpable under the laparoscope, and are thus prone to be missed); and vi) uncontrolled bleeding and injury during the surgery.

The patient survival rate is used as the main measure of the efficacy of treatments for malignant tumors. It is a current aim to achieve an enhanced survival rate following laparoscopy-assisted radical gastrectomy for gastric cancer that is comparable to that of open surgery. In the present study, no significant differences in the 1- and 3-year cumulative survival rates or the mean survival time, were observed between the laparoscopy and the laparotomy groups. With prompt preoperative determination of the indications for surgery, and a detailed intraoperative protocol, the survival time following laparoscopy-assisted D2 radical gastrectomy was similar to that following open surgery, which is consistent with the findings of a previous study (38).

In conclusion, laparoscopy-assisted D2 radical gastrectomy for locally advanced gastric cancer is safe and effective. Provided that the surgeons are experienced at the laparoscopic technique, particularly in D2 radical gastrectomy, and are fully aware of the indications for surgery, laparoscopic D2 radical gastrectomy for gastric cancer may achieve the same mid-term result as the laparotomy, with the additional advantage of minimal invasion. In the present study, the follow-up period was short and the number of cases was small. Therefore, confirmation of the long-term efficacy of laparoscopic D2 radical gastrectomy for gastric cancer remains to be achieved. We propose that, with experience, laparoscopic D2 radical gastrectomy for locally advanced gastric cancer may achieve the same long-term treatment effects as laparotomy.

## Figures and Tables

**Table I:** Comparison of clinical data between patients with distal gastric cancer in laparoscopy and laparotomy groups.

Parameters	Group A	Group B	P-value
Gender			
Male	24	28	0.849
Female	26	34	
Age (years)	61.8±12.17	60.66±13.15	0.419
Ethnicity			
Han	44	54	1.000
Minority	6	8	
Tumor size (cm)	3.83±1.05	3.98±1.17	0.457
Preoperative complications			
Present	24	26	0.569
Absent	26	36	
Method			
Billroth I	32	36	0.564
Billroth II	18	26	
Pathological type			
Mucinous adenocarcinoma, signet ring cell carcinoma and undifferentiated adenocarcinoma	40	39	0.061
Highly/moderately differentiated adenocarcinoma	10	23	
Gross tumor type			
Protruded	30	30	0.256
Ulcerated	20	32	

Group A, laparoscopy; Group B, laparotomy.

**Table II:** Postoperative conditions of patients with distal gastric cancer in laparoscopy and laparotomy groups.

Parameters	Group A	Group B	P-value
Surgery time (min)	251.10±87.38	218.41±60.62	0.046
Intraoperative bleeding volume (ml)^a^	101	210	0.000
Perioperative blood transfusion (fraction of patients)	1/49	9/53	0.023
Distance of the tumor mass from the cut distal end (cm)	3.82±1.11	3.73±1.17	0.791
Incision length (cm)	6.4±0.7	15.6±2.3	0.000
Number of dissected lymph nodes^a^	16.3	17.2	0.435
Time to gastrointestinal function recovery (days)	3.12±0.82	3.8±1.31	0.000
Postoperative complications			
Present	6	18	
Absent	44	44	0.037^b^
Postoperative hospital stay (days)	18.94±7.81	23.61±9.02	0.010^b^

Group A, laparoscopy-assisted surgery; Group B, laparotomy.

a Median for skewed distribution data.
